# The novel sigma-2 receptor ligand SW43 stabilizes pancreas cancer progression in combination with gemcitabine

**DOI:** 10.1186/1476-4598-9-298

**Published:** 2010-11-22

**Authors:** John R Hornick, Jinbin Xu, Suwanna Vangveravong, Zhude Tu, Jonathan B Mitchem, Dirk Spitzer, Peter Goedegebuure, Robert H Mach, William G Hawkins

**Affiliations:** 1 Department of Surgery, Washington University School of Medicine, S. Euclid Avenue, St. Louis, MO, USA; 2 Alvin J. Siteman Cancer Center, Washington University School of Medicine, S. Euclid Avenue, St. Louis, MO, USA; 3 Division of Radiology, Washington University School of Medicine, St. Louis, MO, USA

## Abstract

**Background:**

Sigma-2 receptors are over-expressed in proliferating cancer cells, making an attractive target for the targeted treatment of pancreatic cancer. In this study, we investigated the role of the novel sigma-2 receptor ligand SW43 to induce apoptosis and augment standard chemotherapy.

**Results:**

The binding affinity for sigma-2 ligands is high in pancreas cancer, and they induce apoptosis with a rank order of SV119 < SW43 < SRM *in vitro*. Combining these compounds with gemcitabine further increased apoptosis and decreased viability. Our *in vivo *model showed that sigma-2 ligand treatment decreased tumor volume to the same extent as gemcitabine. However, SW43 combination treatment with gemcitabine was superior to the other compounds and resulted in stabilization of tumor volume during treatment, with minimal toxicities.

**Conclusions:**

This study shows that the sigma-2 ligand SW43 has the greatest capacity to augment gemcitabine in a pre-clinical model of pancreas cancer and has provided us with the rationale to move this compound forward with clinical investigations for patients with pancreatic cancer.

## Background

Pancreatic cancer is the fourth leading cause of cancer related mortalities with an overall five-year survival rate of five percent [1]. Randomized controlled trials have demonstrated modest prolongation of patient survival with chemoradiation or chemotherapy [2-6] and gemcitabine has become standard therapy as a single agent or in combination with other therapies depending on stage [2,5,7]. While results from standard therapies offer some moderate prolongation of survival, novel treatment options are desperately needed. Sigma-2 ligands have been investigated for their therapeutic role in the treatment of cancers, and we have previously shown sigma-2 receptor overexpression in Panc02 tumor bearing C57BL/6 mice and an increased survival in this model by treatment with novel sigma-2 ligands [8,9]. These compounds offer promising potential as novel therapeutics for the treatment of solid tumors, including pancreatic cancer.

Sigma receptors were originally thought to belong to the family of opioid receptors [10], and initial interest was in regard to binding of neuropharmaceuticals such as haloperidol and phenylcyclohexylpiperidine [11,12]. Further study identified two isotypes of the receptor, sigma-1 and sigma-2, with molecular weights of 25 - 29 kDa and 19 - 21.5 kDa respectively [12-15]. While the sigma-1 receptor has been identified and cloned [16,17], the sigma-2 receptor has not been identified. Because of this, studies have revolved around its pharmacological properties. Prototypical compounds for binding studies include [^3^H]-(+)-pentazocine [18,19], with high affinity to sigma-1 and low affinity to sigma-2, and [^3^H]-1,3 di-ortho-tolylguanidine ([^3^H]-DTG), [19] which has equal affinity to both receptors. The concurrent use of non-labeled (+)-pentazocine with [^3^H]-DTG was classically used to study the binding affinity of ligands to the sigma-2 receptor [20] and assisted in their isolation from lipid rafts [21]. Since then, multiple compounds with higher specificity to sigma-2 receptors have been used for binding studies and we have preferred the use of [^3^H]-RHM-1 [22] and [^125^I]-ISO-2 [23] in our laboratory.

Sigma-1 and -2 receptor ligands bind a wide range of normal tissues, but early observations showed sigma-2 receptor over-expression in primary colon cancers, renal carcinomas, and sarcomas [24]. Further studies showed increased expression of sigma receptors in a variety of human and rodent cell lines [25]. Since then, it has been shown that sigma-2 receptors are upregulated in solid tumors and that their presence can be used as a marker of proliferation, making them an attractive target for imaging of tumors *in vivo *[26]. In addition, multiple studies have shown that several different sigma-2 ligands induce tumor selective cytotoxicity and apoptosis, the mechanism of which is currently poorly understood [9,27-30].

We have identified a sigma-2 ligand, SW43, similar in structure to the previously studied SV119, with enhanced activity. We have previously shown that SV119 specifically binds to sigma-2 receptors and induces apoptosis in pancreas cancer [8,9]. In this study, we systematically tested sigma-2 ligands *in vitro *and *in vivo *for relative effectiveness in pancreatic cancer and their relative toxicity in order to identify the best candidate to move into a clinical trial.

## Results

### Sigma-2 ligands have high affinity for pancreas cancer and decrease viability

Several groups have shown that sigma-2 specific ligands decrease viability in cancer cells [27,28,30] and we have reported that sigma-2 specific ligands induce apoptosis in pancreas cancer [9]. In this study, we compared the antitumor effects of the novel sigma-2 receptor ligand SW43 with our previously studied compound, SV119, and a commercially available sigma-2 ligand, siramesine (SRM).

**Figure 1:**
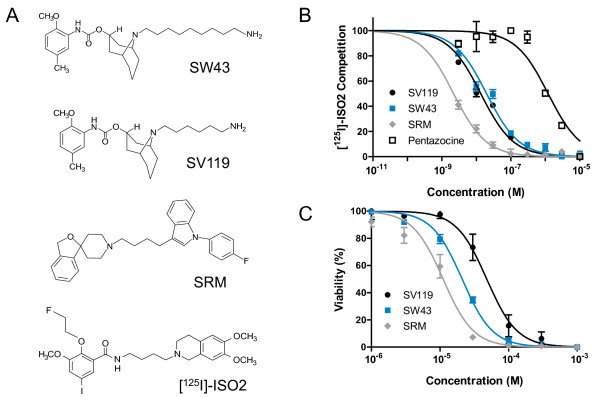
**Sigma-2 ligands have high affinity for pancreas cancer and decrease viability. **A. Chemical structures of sigma-2 ligands. B. Representative competitive binding data for inhibition of [^125^I]-ISO-2 binding to sigma-2 receptors in Panc02 tumor membrane homogenates. IC_50 _for binding affinities are pentazocine 1381 ± 33 nM, siramesine 1.9 ± 0.1 nM, SV119 7.8 ± 1.7 nM, and SW43 18 ± 2.1 nM. C. Pancreas cancer cell lines were treated with escalating doses of the sigma-2 ligands SV119, SW43, and siramesine (SRM) or DMSO vehicle for 18 hours and viability relative to vehicle determined by CellTiter-Glo Luminescent Viability Assay (Promega).

The sigma-2 specific ligands SRM and SV119 and sigma-1 specific ligand pentazocine were compared to the novel sigma-2 ligand SW43 in characterization of the pharmacological binding profile to sigma-2 receptors in Panc02 tumor membrane homogenates. Competitive binding curves (Figure 1B) show the affinity rank order for sigma ligands as Siramesine > SV119 ≥ SW43 >> (+)-pentazocine. The IC_50 _values (mean ± SEM) for inhibiting [^125^I]-ISO-2 binding to sigma-2 receptors are pentazocine 1381 ± 33 nM, SRM 1.9 ± 0.1 nM, SV119 7.8 ± 1.7 nM, and SW43 18 ± 2.1 nM.

Multiple human (AsPC1, BxPC3, Cfpac, Panc1, and MiaPaCa-2) and mouse (Panc02) (Figure 1C) pancreas adenocarcinoma cell lines were treated with doses of sigma-2 ligand ranging from 1 to 1000 μM for 18 hours. To compare the relative effect on viability, IC_50 _values for each cell line were calculated (Table 1). The range of IC_50 _values was 50-106 μM for SV119, 21-56 μM for SW43, and 11-36 μM for SRM. From these IC_50 _values, we observe that the relative cytotoxicity was SRM > SW43 > SV119.

**Table 1:** IC_50 _(μM) for pancreas cancer cell lines treated with sigma-2 ligands for 18 hours.

Cell Line	SV119	SW43	SRM
Panc02	50 ± 9	21 ± 0	11 ± 2
MiaPaCa2	58 ± 8	29 ± 6	17 ± 1
Panc1	64 ± 10	35 ± 3	19 ± 2
Bxpc3	84 ± 17	38 ± 2	19 ± 3
Cfpac	76 ± 14	49 ± 4	16 ± 1
Aspc1	106 ± 7	56 ± 1	36 ± 8

(Mean ± SEM), n ≥ 3

### Sigma-2 ligands induce caspase-3 activation

**Figure 2:**
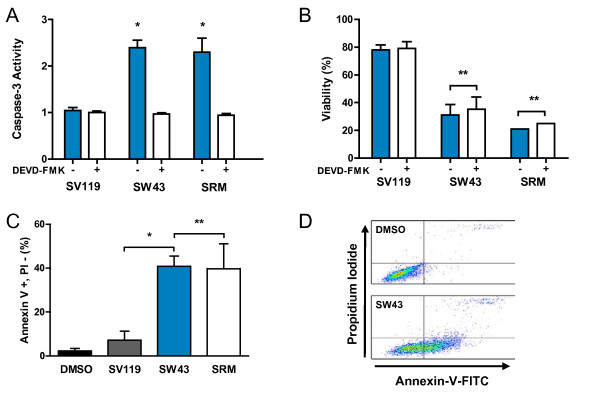
**Sigma-2 ligands induce caspase-3 activation in pancreas cancer. **Panc02 cells were pre-treated with caspase-3 inhibitor (DEVD-FMK 1 μM) or DMSO vehicle for one hour prior to exposure to sigma-2 ligands (25 μM) SV119, SW43, siramesine (SRM) or DMSO vehicle for 18 hours. A. Caspase-3 activation was quantified by cleavage of the fluorogenic substrate Ac-DEVD-AMC and expressed relative to vehicle. The caspase-3 specific inhibitor DEVD-FMK abrogates caspase-3 dependent cleavage. B. Caspase-3 inhibition does not protect cells from sigma-2 ligand induced cell death. C. Flow cytometry for Annexin-V verifies apoptosis by sigma-2 ligand. D. Representative flow cytometry dot plot showing cells moving from Annexin-V negative quadrant (upper panel) with vehicle towards Annexin-V positive quadrant (lower panel) with SW43 treatment. (Means ± SEM), n ≥ 3, *p < 0.05, **p > 0.05.

Sigma-2 ligands have been observed to induce apoptosis by caspase-3 dependent and independent mechanisms in several cancer cell lines [9,28,31]. To compare caspase-3 activity induced by SV119, SW43, and SRM, Panc02 mouse adenocarcinoma cells were treated with sigma-2 ligands (25 μM) for 18 hours and assayed for cleavage of the caspase-3 fluorogenic substrate Ac-DEVD-AMC. To determine whether this activity was caspase-3 specific, the caspase-3 inhibitor DEVD-FMK (1 μM) was applied one hour prior to sigma-2 ligand. SW43 and SRM similarly increased caspase-3 activity by 2.4 and 2.3 fold (p < 0.001) respectively compared to cells treated with DMSO vehicle only, while SV119 had little effect on caspase-3-like activity (p > 0.05) under these conditions (Figure 2A). Treatment with DEVD-FMK did not change the effect on viability (Figure 2B) (p > 0.05). To verify that caspase-3 activity correlates with apoptosis, cells were also assayed for extracellular Annexin-V expression (Figure 2C, D). Expression was increased from 2 ± 1 percent with vehicle to 7 ± 4 percent for SV119 (p > 0.05), 40 ± 5 percent for SW43 (p < 0.05), and 40 ± 11 percent for SRM (p < 0.05).

### Sigma-2 ligands generate reactive oxygen species

**Figure 3:**
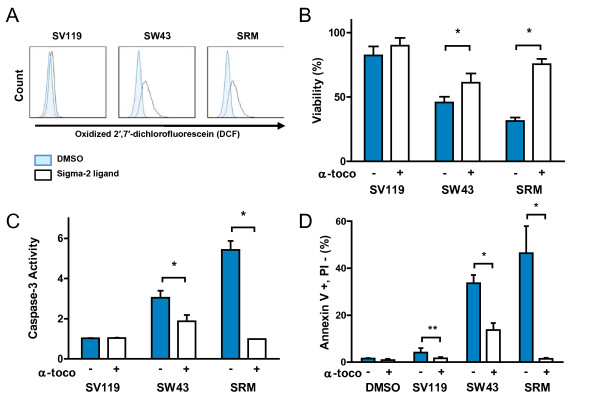
**Sigma-2 ligands induce generation of reactive oxygen species in Panc02 cells. **A. Sigma-2 ligands induce oxidation of 5-(and-6)-carboxy-2',7'-dichlorodihydro-fluorescein diacetate (carboxy-H_2_DCFDA) to 2',7'-dichlorofluorescein (DCF) and quantified by fluorescence of DCF. B. Viability was partially rescued for SW43 and siramesine (SRM) in the presence of alpha-tocopherol (200 μg/mL), but not for SV119. C. Sigma-2 ligand induced caspase-3 activity was partially reduced for SW43 in the presence of lipophilic α-toco, but completely for SRM. D. Flow cytometry for Annexin-V verifies that caspase-3 activity correlates with apoptosis. (Means ± SEM), n ≥ 3, *p < 0.05, **p > 0.05.

The mechanism of sigma-2 ligand induced apoptosis is not well understood and has been shown in different cells types to be caspase-3 dependent or independent [9,31,32]. While we have previously shown that sigma-2 ligands can induce caspase-3 in pancreatic cancer cells [8,9], others have shown SRM increases reactive oxygen species (ROS) [28,29]. In order to compare these sigma-2 ligands, we quantified the capacity of SV119, SW43, and SRM to generate ROS. Panc02 were treated with sigma-2 ligands (25 μM 18 hrs) or vehicle (Figure 3) and incubated with 5-(and-6)-carboxy-2',7'-dichlorodihydro-fluorescein diacetate (carboxy-H_2_DCFDA) Image-iT Live Green Reactive Oxygen Species Detection Kit (Molecular Probes, Eugene, OR) for analysis by flow cytometry. The oxidation of carboxy-H_2_DCFDA to 2',7'-dichlorofluorescein (DCF) leads to detectable fluorescence in the FL1channel. We observed that SV119 treatment generated little ROS, while SW43 and SRM generated a large amount of ROS (Figure 3A). In order to determine whether viability could be rescued with the lipid antioxidant alpha-tocopherol (α-toco), cells were pretreated with α-toco (200 μg/mL) for one hour prior to sigma-2 ligand treatment. Viability was partially rescued for SW43 (45% to 61%, p < 0.05) and SRM (32% to 76%, p = 0.01) in the presence of α-toco, but not for SV119 (82% to 90%, p > 0.05) (Figure 3B). To establish whether the rescue of viability by α-toco was through protection against apoptosis, we quantified the caspase-3 activity and Annexin-V staining (Figure 3C and 3D). We observed that sigma-2 ligand induced caspase-3 activity was partially reduced for SW43 (3 fold down to 1.9 fold, p < 0.01) in the presence of lipophilic α-toco, but was completely reduced for SRM (5.4 fold down to 0.98 fold, p = 0.01). Flow cytometry analysis for Annexin-V verified that induction of caspase-3 activity correlated with apoptosis (Figure 3D). Apoptosis by SW43 was decreased from 34% to 14%, p < 0.05, and SRM-induced apoptosis decreased from 46% to 1.5%, p < 0.05.

### Sigma-2 ligands enhance standard chemotherapy by increasing apoptosis

**Figure 4:**
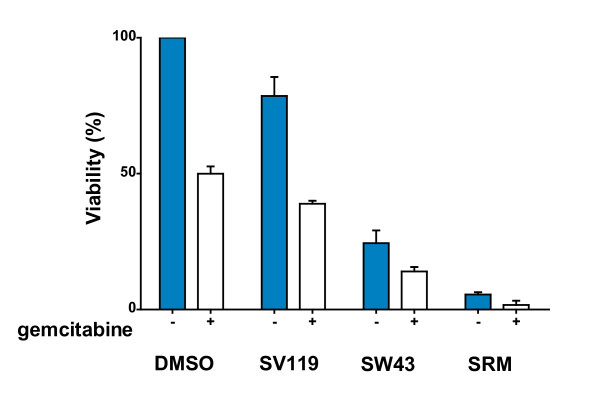
**Sigma-2 ligands enhance gemcitabine induced cell death in pancreas cancer. **Multiple pancreatic cancer cell lines, including Cfpac, Panc1, BxPC3 (Table 1), and Panc02 (shown) were treated with DMSO vehicle or sigma-2 ligands (25 μM) SV119, SW43, or siramesine (SRM) following 24 hour pre-treatment with gemcitabine (0.5 μM). Eighteen hours later cell viability was determined using the CellTiter-Glo Luminescent Viability Assay (Promega), (mean ± SEM), n ≥ 3.

We have previously reported that sigma-2 ligands potentiate chemotherapy for pancreatic cancer *in vitro *and *in vivo *(9). Here, we compared viability after SW43 treatment in selected human (Panc1, BxPC3, and Cfpac) and mouse (Panc02) (Figure 4) pancreas adenocarcinoma in combination with gemcitabine. These cells have sufficient variability in sensitivity to gemcitabine to detect overlapping mechanism of apoptosis by gemcitabine or sigma-2 ligand. Cells were treated with gemcitabine (500 nM) or vehicle for 24 hours prior to sigma-2 ligand (25 μM) treatment for 18 hours. Gemcitabine alone decreased viability in our cell lines (range 40 to 71%, Table 2) compared to vehicle and sigma-2 ligands were additive to that of gemcitabine in each cell line. To determine whether decrease in viability was through enhancement of apoptosis (Additional File 1), Panc02 cells were stained with Annexin-V-FITC conjugated antibody and propidium iodide (PI). Annexin-V positive/PI negative cells represent early apoptotic cells and Annexin-V positive/PI positive cells represent late apoptotic and necrotic cells. We observed a shift through the early apoptotic population towards late apoptotic as the relative strength of the treatment increased. Enhancement of gemcitabine by SW43 was greater than that of the previously reported compound SV119 (p < 0.01).

**Table 2:** Viability of pancreas cancer cell lines following treatment with sigma-2 ligands and gemcitabine.

Gemcitabine	-	+	-	+	-	+	-	+
**Cell Line**	**DMSO**		**SV119**		**SW43**		**SRM**	

Panc02	100	50 ± 3	79 ± 7	39 ± 1	24 ± 5	14 ± 2	6 ± 0	2 ± 2
Panc1	100	47 ± 5	81 ± 6	41 ± 5	43 ± 5	19 ± 4	8 ± 4	3 ± 3
BxPC3	100	71 ± 1	66 ± 2	52 ± 1	43 ± 4	33 ± 4	8 ± 2	2 ± 1
Cfpac	100	40 ± 8	88 ± 4	40 ± 4	60 ± 6	21 ± 7	8 ± 5	4 ± 2

Pancreas cancer cell lines were pretreated with gemcitabine (0.5 uM) or vehicle for 24 hours followed by sigma-2 ligand (25 uM) treatment for 18 hours and viability represented as percentage relative to vehicle, (mean ± SEM), n ≥ 3.

### Sigma-2 ligands confer a protective effect against pancreas cancer *in vivo*

**Figure 5:**
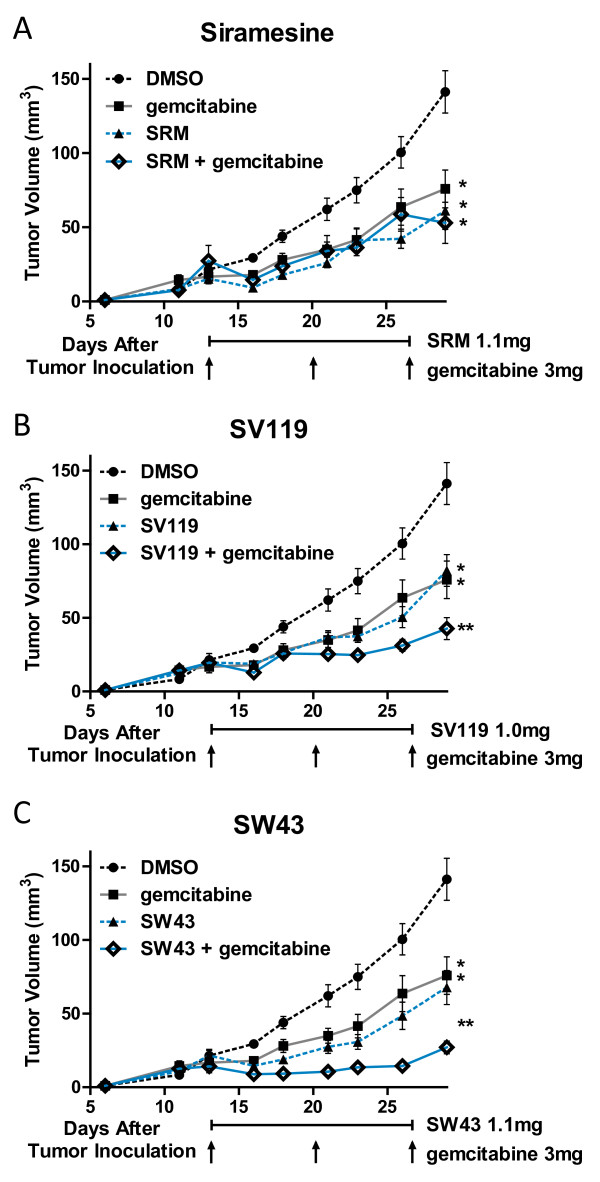
**Sigma-2 ligands decrease Panc02 tumor burden in C57BL/6. **One million Panc02 cells were inoculated subcutaneously into female, 10 week old C57BL/6 mice and when tumors had reached a mean diameter of 5 mm, daily sigma-2 ligand treatment with or without weekly gemcitabine began by IP injection. All treatments decreased tumor volume compared to DMSO vehicle alone, p < 0.001. A. Siramesine (SRM) treatment did not confer a significantly different tumor volume than gemcitabine alone, *p > 0.05. Single agent sigma-2 ligands SV119 B. and SW43 C. decreased tumor volume similar to gemcitabine, *p > 0.05, while combination therapy produced much smaller tumors, **p < 0.001.

We have previously shown that sigma-2 ligands augment the antitumorigenic effect of gemcitabine *in vivo *[9]. Here, we compared the novel compound SW43 against SV119 and SRM in combination therapy with gemcitabine. C57BL/6 female mice were inoculated with 1 × 10^6 ^Panc02 cells subcutaneously and 13 days following tumor injection, when all mice had tumors with a diameter of approximately 5 mm, mice were randomized into groups of n = 12-15. Sigma-2 ligands SW43 (1.1 mg), SV119 (1.0 mg), or SRM (1.1 mg) were given by IP injection daily for two weeks with or without weekly gemcitabine (3.0 mg). (Figure 5) Following conclusion of treatment (Day 27), tumors were significantly smaller for individual treatment groups SW43 (mean = 67 mm^3^) and gemcitabine (mean = 76 mm^3^) compared to vehicle (mean = 141 mm^3^) (p < 0.001). Single agent sigma-2 ligand treated mice had similar tumor volumes that were statistically similar to gemcitabine. However, mice receiving combination therapy of SW43 and gemcitabine had the smallest tumors (mean = 27 mm^3^) (p < 0.001) that, over the entire two week treatment course, stabilized and tumor size on day 13 was equal to tumor size on day 27. Combination with gemcitabine decreased tumor volume further for SW43 (mean = 27 mm^3^) compared to SV119 (mean = 43 mm^3^) or SRM (mean = 53 mm^3^) (Figure 5C,A, and 5B, respectively). Even though treatment was discontinued on day 27, sigma-2 ligands also conferred a survival advantage for mice in this trial. The median survival for mice treated with DMSO was 46 days compared to 57 days for mice treated with a combination of SW43 and gemcitabine (p < 0.001). All other treatment groups had a median survival of 49 to 52 days. One animal from the SW43 and gemcitabine combination group had complete regression of the tumor (data not shown). The initial tumor volume in this mouse did not differ from the mean of cohorts until two weeks following treatment (day 42). At this time, the tumor steadily decreased in volume until it was no longer palpable by day 51. The local area of tumor growth was surgically removed when the tumor was no longer palpable after two repeated measurements, and histologic examination confirmed the absence of tumor.

### Sigma-2 ligands have minimal toxicity *in vivo*

Therapeutic window is a concern in treating patients with chemotherapies. We have previously performed toxicity profiling for SV119 *in vivo *and found toxicity to be negligible [8]. Equimolar amounts of SW43 (1.1 mg) and SV119 (1.0 mg) were compared in C57BL/6 female mice by daily treatment for two weeks, with and without weekly gemcitabine (3 mg). Mice were sacrificed for blood cytology and serum chemistries (Additional File 2), and organs for histologic and gross pathology review. Of note, we observed minor anemia and decreased pancreatic enzyme levels that were statistically different p < 0.05, but not clinically significant. When combined with gemcitabine, hemoglobin levels decreased 2.7 g/dL for SV119, 3.7 g/dL for SV119, and 4.9 g/dL for SW43. Gross pathology and histology reports of brain, kidney, liver, and pancreas were not appreciably different from untreated control animals (data not shown). Mice appeared well throughout treatment and did not experience any treatment related deaths.

## Discussion

Pancreatic cancer is an aggressive disease and current therapy is disappointing. Many tumors are inoperable at the time of diagnosis, and recurrence is high in those deemed operable. Therefore, many patients in clinical trials have received chemotherapy or radiation therapy in different treatment combinations, as neo-adjuvants or as an adjuvant following surgery. While the technology of therapeutic options has increased over the years, the long-term survival rate has changed little over the past few decades, stressing the need for more effective treatments. We have shown that sigma-2 ligands increase survival of tumor bearing mice and are additive to combination regimens with chemotherapies. In this study, we identified a novel sigma-2 ligand that has superior efficacy in pancreatic cancer with limited offsite toxicity.

Chemotherapies and radiation are common in that they disregulate the cell cycle directly or indirectly, and activate apoptosis of tumor cells. Without specific targeting of these treatments, systemic toxicities occur, decreasing the therapeutic window. Compounds which target sigma-2 receptors appear to be selective to cancers. While, sigma-1 and sigma-2 receptors are located in many normal tissues, sigma-2 receptors are highly expressed in tumor cells, allowing selective targeting. We have also shown sigma-2 receptor ligands specifically taken up in a mouse model of pancreas cancer by micro PET/CT imaging [8]. This specificity may also allow targeted therapy by the delivery of pro-apoptotic molecules specifically to pancreatic cancer cells.

In this study, we identified a novel sigma-2 receptor ligand that has superior efficacy in pancreatic cancer. Multiple human and mouse pancreatic adenocarcinoma cell lines were tested *in vitro *to asses the effect of our compounds on viability. We showed that SW43 had a greater capacity to decrease viability compared with SV119 in all pancreas cancer cell lines tested, with an IC_50 _value at least half that of SV119. Sigma-2 ligands also had an additive effect with gemcitabine to induce cell death. Cell death by SW43 occurs through a mechanism consistent with apoptosis in pancreatic cancer cell lines and induces caspase-3 activity even greater than our previous compound SV119, though caspase-3 inhibitor could not prevent cell death. The SW43 effect *in vitro *was similar to that of siramesine, a widely studied sigma-2 ligand. We verified that increased caspase-3 activity correlated with induction of apoptosis by flow cytometry for Annexin-V binding and the apoptotic effect was even further increased by pre-treatment with gemcitabine.

We chose siramesine for comparison in our study because it is an established sigma-2 ligand that has been used in a phase I clinical trial as an anxiolytic [33]. Although siramesine did not meet the objective clinical response in this trial, it was shown to be well tolerated in 200 healthy volunteers. Following that trial, siramesine has been tested in mice for the treatment of breast cancer and was found to induce cytotoxicity mediated in part by oxidative stress [28,32]. It was not determined whether this effect was mediated by sigma-2 receptors or by siramesine interactions in the membranes, though siramesine has been recently shown to form high-affinity complexes with phosphatidic acid [34].

The generation of reactive oxygen species (ROS) has been well established to be a by-product as well as an initiator of apoptosis and necrosis. Intracellular transmission of apoptotic signals occurs through production of ceramide or directly acting on the mitochondria [35]. Depending on cell type, ROS have been shown to increase mRNA and protein expression of pro-apoptotic molecules such as FasL, FasR, Bax, and caspases-2 and -3, modulation of MAPK pathways, as well as release of cytochrome c from the mitochondria [36,37]. Mechanisms of apoptosis by ROS are varied and may be cell-type dependent.

We observed that SW43 induces ROS in pancreas cancer cells and that apoptosis can be partially relieved by treatment with the antioxidant alpha-tocopherol. In contrast, apoptosis by siramesine induced ROS was completely blocked by alpha-tocopherol, while SV119 generated little ROS. This suggests that the mechanism of SW43 induced apoptosis is at least partially through the generation of ROS. Based on the similar structure and binding characteristics of SW43 and SV119, it is plausible that they share an apoptotic pathway independent of siramesine.

In Panc02 cells, binding affinity is high for sigma-2 and low for sigma-1, with the relative affinity for sigma-2 ligands being SRM > SW43 = SV119. Increased binding affinity of siramesine supports our data showing a lower IC_50 _for viability than SW43 and SV119 and suggests that sigma-2 receptor binding is important for initiation of apoptosis. Binding affinity does not explain the lower IC_50 _for SW43 versus SV119 though. We speculate that the extended aminoakyl chain of SW43 increases lipophilicity and enhances membrane diffusion into the cell. Evidence for membrane diffusion is supported by our previous studies showing that sigma-2 receptor ligand internalization is only partially blocked with the well studied endocytosis inhibitor phenylarsine oxide [38].

To verify that these promising *in vitro *results translate into an anti-tumorigenic effect *in vivo*, we utilized the aggressive Panc02 cell line in C57BL/6 mice. We have shown that this treatment schedule resulted in minimal toxicity *in vivo *that was well tolerated. Daily treatment of sigma-2 ligands produced an effect statistically similar to gemcitabine alone. Though SRM showed superior efficacy *in vitro*, the results did not translate in our *in vivo *model. SW43 combination therapy with gemcitabine resulted in the smallest tumor volume which had stabilized during the two week treatment period. As well, we demonstrate a therapeutic drug combination that completely eradicated an established Panc02 tumor in a C57BL/6 mouse, which we have not observed in over a decade of our own experience. Survival was increased for the combination of SW43 with gemcitabine from this treatment course. We expect that based on the stabilization of tumor volume during the limited treatment period (2 weeks), survival would be increased if the treatment duration was lengthened.

## Conclusions

We have shown that SW43 has high binding affinity for sigma-2 binding sites in pancreatic cancer cell lines and treatment of a variety of pancreas cancer cell lines with SW43 showed higher cytotoxicity than our previous compound SV119. As well, SW43 augmented the effects of gemcitabine *in vitro *and *in vivo*. The data presented here supports SW43 as the most promising sigma-2 ligand for clinical development in the treatment of pancreatic cancer.

## Methods

### Cell Culture

The human pancreas cancer cell lines BxPC3, AsPC1, Cfpac, Panc1, and MiaPaCa-2 were obtained from ATCC (Bethesda, MD). Murine pancreas adenocarcinoma Panc02 was a gift from Bryan Clary (Duke University). Panc02, AsPC1, and BxPC3 cells were maintained in RPMI (GIBCO) supplemented with L-glutamine (2 mM), HEPES (1 mM), pyruvate (1 mM), sodium bicarbonate (0.0075% w/v); Cfpac in IMDM containing L-glutamine (2 mM) and HEPES (25 mM); Panc1 and MiaPaCa in DMEM (GIBCO) containing D-glucose (4.5 g/L), L-glutamine (2 mM), HEPES (25 mM). All medias were supplemented with penicillin and streptomycin (100 IU/mL), amphotericin (0.25 μg/mL), and 10% FBS (Atlanta Biologicals, Lawrenceville, GA). Cells were seeded at a concentration of 1.5 × 10^6^/mL unless otherwise noted and maintained in a humidified atmosphere of 5% CO_2 _at 37°C.

### Compounds

Sigma-2 ligands were synthesized as previously described [28]. Chemical synthesis of these ligands is based off a molecule with a 9-azabicyclo[39]nonoan-3β-yl ring with an aminoalkyl extension (Figure 1A). Sigma-2 selectivity is increased by greatly decreasing the specificity to sigma-1 receptors [39]. SW43 is similar in structure to SV119, with the aminoalkyl extension lengthened from six carbons to ten carbons. [^125^I]-ISO-2 was prepared by an iododestannylation reaction of the corresponding tributyltin precursor [23]. The caspase-3 inhibitor Z-DEVD-FMK was obtained from R&D Systems (Minneapolis, MN); the caspase-3 cleavage substrate Ac-DEVD-AMC from Bachem Biosciences, Inc (King of Prussia, PA); gemcitabine from Eli Lilly and Company (Indianapolis, IN). All other reagents were obtained from Sigma (St Louis, MO) unless otherwise stated. Sigma-2 ligands and inhibitors were dissolved in DMSO and treatments *in vitro *and *in vivo *received DMSO at a final concentration less than 0.3%.

### Competitive binding analysis

Sigma-2 receptor competition was performed with [^125^I]-ISO-2, a benzamide analog of [^3^H]-RHM-1, and was used for this study due to higher specificity to sigma-2-receptor ([^125^I]-ISO-2, sigma-2 = 0.26 nM, sigma-1 = 2,150 nM, sigma-1:sigma-2 ratio = 8,269) compared to ([^3^H]-RHM-1, sigma-2 = 10.3 nM, sigma-1 = 3078, sigma-1:sigma-2 ratio = 299) [23]. Membrane homogenates were prepared from ~300 mg Panc02 tumor allografts, as previously described in our group [8]. ~100 μg membrane homogenates were diluted with 50 mM Tris-HCl buffer, pH 8.0 and incubated with ~0.5 nM radioligand [^125^I]-ISO-2 in a total volume of 150 µl at 25°C in 96 well polypropylene plates (Fisher Scientific, Pittsburgh, PA). After incubation of 30 min, the reactions were terminated by the addition of 150 µl of cold wash buffer (10 mM Tris-HCl, 150 mM NaCl, pH 7.4, at 4°C) using a 96 channel transfer pipette (Fisher Scientific, Pittsburgh, PA), and the samples harvested and filtered rapidly to 96 well fiber glass filter plate (Millipore, Billerica, MA). Each filter was washed with 200 µl of ice-cold wash buffer for a total of three washes. Then the filters were punched out and a Packard gamma counter (Beckman, Fullerton, CA) with a counting efficiency of 75% for ^125^I was used to quantitate the bound radioactivity. Nonspecific binding was determined from samples which contained 10 µM haloperidol.

Data from the competitive inhibition experiments were modeled using nonlinear regression analysis to determine the concentration of inhibitor that inhibits 50% (IC_50_) of the specific binding of the radioligand (IC_50 _value). The competition curves were modeled for a single site using the following equation:

$$B_{s}=B_{0}-\left[B_{0}*I/\left(IC_{50}+I\right)\right]$$

Where *B*_s _is the amount of the radioligand bound specifically to the membrane homogenates (i.e., *B*_s _= *B*_t _- *B*_ns_, where *B*_t _is the total bound radioactivity and *B*_ns _is the nonspecific binding of the radiotracer), *B*_0 _is the amount of the radioligand bound in the absence of the competitive inhibitor, *I is *the concentration of the competitive inhibitor and the IC_50 _is the concentration of competitive inhibitor that blocks 50% of the total specific binding. The data analysis and curve fitting for IC_50 _were performed with the KaleidaGraph software purchased from Synergy (Reading, PA) [22,23].

### Detection of cell viability *in vitro*

Pancreas cancer cell lines maintained at optimal culture conditions were seeded into 96 well plates the day prior to treatment with escalating doses of the sigma-2 ligand. Additionally, cells were pretreated with gemcitabine (500 nM) for twenty four hours prior to sigma-2 ligand treatment (25 μM). Eighteen hours after treatment, cell viability was determined using the CellTiter-Glo Luminescent Viability Assay from Promega (Madison, WI). This assay quantitates the number of metabolically active cells by detecting ATP and gives a linear correlation of viable cells to luminescence. The viability assay was conducted essentially as described by the manufacturer and luminescence quantified using a SpectraMax Gemini microplate spectrofluorometer from Molecular Devices (Silicon Valley, CA). Viability is expressed relative to vehicle. Viability versus concentration of sigma-2 ligand was fit by non-linear regression and the IC_50 _at 18 hours determined. Experiments were conducted with duplicate wells and conducted at least three times.

### Detection of caspase-3 activity *in vitro*

Caspases are members of a family of cysteine proteases with aspartate specificity that play a role in apoptosis. The enzymatic substrate for caspase-3, found within poly(ADP-ribose) polymerase (PARP), DEVD216-G217, is utilized to observe activated caspase-3 by the liberation of the fluorogenic molecule 7-amino-4-methylcoumarin (AMC) from DEVD [40-42]. Murine pancreas adenocarcinoma cell line Panc02 was maintained at optimal culture conditions and seeded in 96 well plate as above. The following day, cells were pretreated for one hour with the caspase-3 inhibitor Z-DEVD-FMK (1 μM) or vehicle. Cells were then treated with sigma-2 ligands (25 μM) or vehicle and assayed 18 hours later. A 5X assay buffer containing EDTA (10 mM), CHAPS (5%), HEPES (100 mM), DTT (25 mM), and Ac-DEVD-AMC (250 μM) was added directly to the cell media and incubated for two hours at 37°C on a microplate shaker before detection of liberated AMC. The fluorescence of AMC was detected using a SpectraMax Gemini microplate spectrofluorometer, Molecular Devices (Silicon Valley, CA) with excitation at 355 nm and emission at 450 nm.

### Apoptosis detection by flow cytometry for Annexin-V

Annexin-V has a high affinity for phosphatidylserine and is used as a marker of apoptosis [43]. Panc02 cells were seeded into 12 well plates and the following day treated with sigma-2 ligand (25 μM) or vehicle. Eighteen hours later the cells were assayed using the Annexin-V FITC Kit from Invitrogen Corporation (Camarillo, CA). The cells were prepared as directed by the manufacturer and analyzed with a FACSCalibur flow cytometer (BD Biosciences, San Jose, CA).

### Detection of reactive oxygen species by flow cytometry

Panc02 cells were seeded into 12-well plates one day prior to treatment with sigma-2 ligand (25 μM). Eighteen hours later cells were stained with 25 μM 5-(and-6)-carboxy-2',7'-dichlorodihydro-fluorescein diacetate (carboxy-H_2_DCFDA) (Image-iT Live Green Reactive Oxygen Species Detection Kit, Molecular Probes, Eugene, OR) for 30 minutes at 37°C and washed once with PBS before analysis with a FACSCalibur flow cytometer (BD Biosciences, San Jose, CA). The oxidation product of carboxy-H_2_DCFDA to 2',7'-dichlorofluorescein (DCF) fluoresceses with an emmission maximum of 529 nm and was detected in the FL1 channel.

### Tumor growth, survival, and toxicity *in vivo*

In this pre-clinical model, we utilized the Panc02 cell line [44], which is weakly immunogenic in C57BL/6 mice and aggressive with a subcutaneous inoculum of 1 × 10^5 ^cells being lethal within 6-8 weeks. Female, 10 week old C57BL/6 mice, obtained from Harlan Laboratories, Inc (Indianapolis, IN), were injected subcutaneously in the right flank with 1 × 10^6 ^Panc02 cells suspended in 200 μL non-supplemented RPMI. When tumors had reached approximately 5 mm in diameter, treatment began with IP injection of DMSO or equimolar mass of the sigma-2 ligands SV119 (1 mg), SW43 (1.1 mg), or SRM (1.1 mg) daily for two weeks, with or without once weekly gemcitabine (3 mg) for four weeks. Blood was collected from several mice by intracardiac withdrawal for cytologic (complete blood count) and biochemical analysis (AST, ALT, BUN, Cr, amylase, and lipase) by the Washington University Department of Comparative Medicine. Organs were examined grossly and histologically. Tumors were measured three times weekly with digital callipers and the volume calculated using the equation V = d_1_(d_2_^2^)/2, (V = volume, d_1_= larger diameter, d_2_= smaller diameter). All mice were euthanized when tumors reached a diameter of 15 mm or had ulcerated. Studies were performed in accordance with the animal studies protocol approved by the Washington University Institutional Animal Care Facility.

### Statistical Analysis

The Washington University Division of Biostatistics was consulted for statistical analysis. Data plotting and statistical analysis was conducted using GraphPad Prism (GraphPad Software, San Diego, CA). Data in figures represent the mean ± SEM. Viability IC_50 _values at 18 hours were calculated by line fitting normalized viability versus concentration with non-linear regression and statistical significance determined using one-way ANOVA. Differences in viability, caspase-3 activity, apoptosis, and oxidation status were analyzed using two-way ANOVA to identify differences and confirmed with paired two-tailed t-tests. Blood cytology and biochemistry results were analyzed using one-way ANOVA with Tukey's multiple comparison test. Statistical analysis for the difference in tumor volume between treatments groups was determined with the repeated measures ANOVA. Kaplan-Meier survival curves were plotted and differences compared with a log-rank test. A p-value of less than 0.05 was considered significant for all tests.

## Competing interests

The authors declare that they have no competing interests.

## Authors' contributions

JRH performed assays for viability and apoptosis, treatment and toxicity monitoring of mice, and drafted the manuscript. JX evaluated the pharmacologic profile. SV conducted chemical synthesis of compounds. ZT conducted chemical synthesis of compounds. JBM was involved in design and assistance of experiments. DS was involved in design of experiments and critical review of manuscript. PG was involved in design of experiments and critical review of manuscript. RHM was involved in conception of compounds and critical review of manuscript. WGH was involved in conception of the use of compounds in pancreas cancer, design of experiments, and critical review of manuscript. All authors have read and approve the final manuscript.

## Supplementary Material

Additional file 1 **Sigma-2 ligands enhance gemcitabine induced apoptosis evidenced by detection of Annexin-V by flow cytometry. **Panc02 cells were pre-treated with gemcitabine (500 nM) or vehicle for 24 hours prior to exposure to sigma-2 ligands (25 μM) or vehicle for 18 hours. Cells were then stained with Annexin-V-FITC conjugated antibody and propidium iodide (PI) to be assessed by flow cytometry. Sigma-2 ligands induced apoptosis alone and enhanced gemcitabine induced apoptosis. Annexin-V+/PI- represent early apoptotic cells and Annexin-V+/PI+ cells show late apoptotic cells. Data is representative of triplicate experiments. Click here for file

Additional file 2 **Blood cytology and serum chemistries for C57BL/6 mice treated daily with sigma-2 ligand and/or weekly gemcitabine for 2 weeks. **Blood cytology was analyzed for white blood count (CBC), hemoglobin (HGB), and platelets (PLTS), while serum chemistries were analyzed for the liver enzymes aspartate aminotransferase (AST) and alanine aminotransferase (ALT), renally cleared metabolites blood urea nitrogen (BUN) and creatinine (CR), as well as pancreatic enzymes amylase and lipase. Of statistical and clinical significance was a decrease in HGB in groups receiving combination treatment. Click here for file
