# Supplementary figures and images for: The novel sigma-2 receptor ligand SW43 stabilizes pancreas cancer progression in combination with gemcitabine
Source: Mol Cancer. 2010 Nov 22;9:298. doi: 10.1186/1476-4598-9-298 (PMC3106998; doi:10.1186/1476-4598-9-298)

## Slide 1
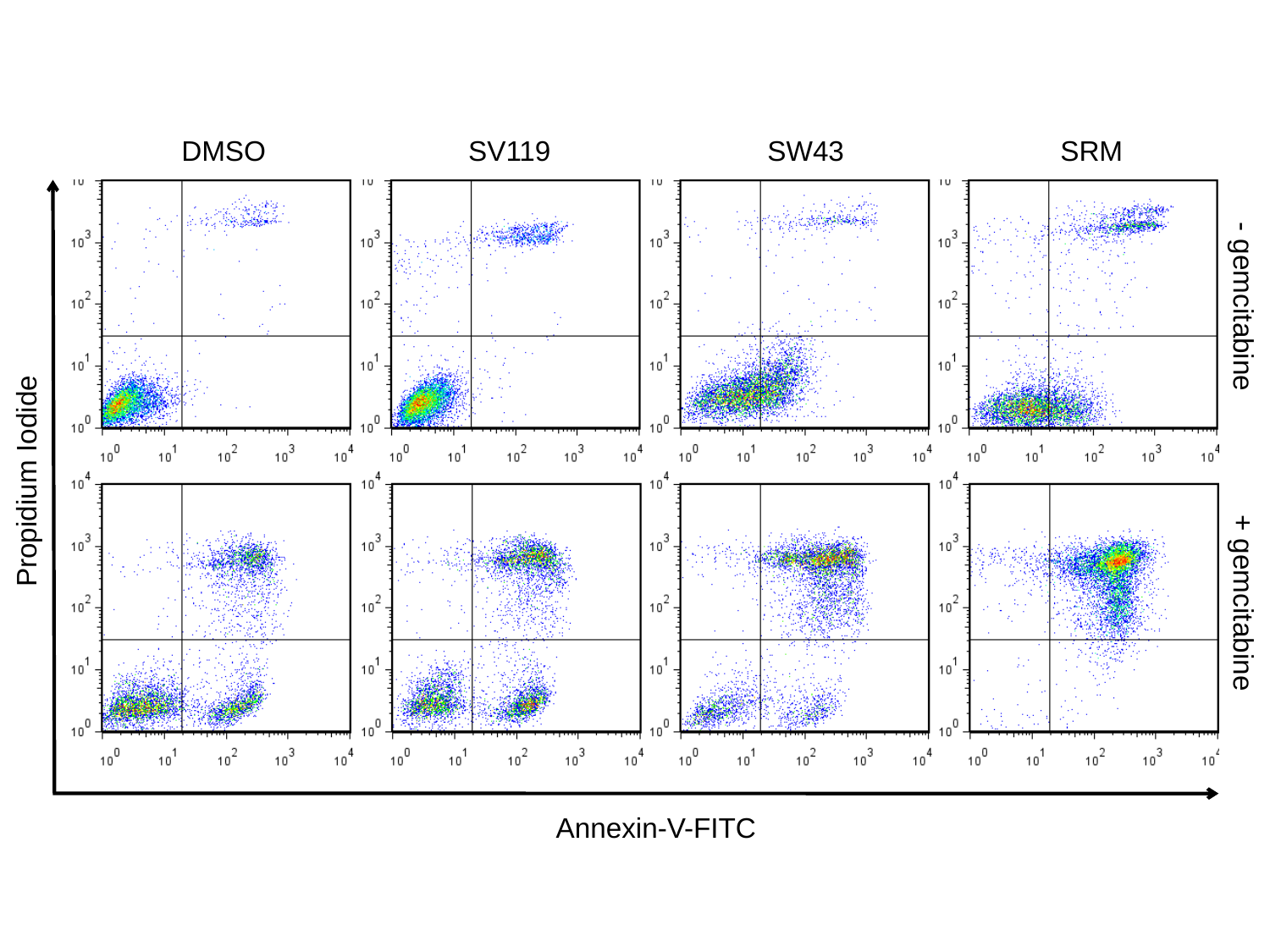

DMSO
SV119
SW43
SRM
- gemcitabine
Propidium Iodide
+ gemcitabine
Annexin-V-FITC

Supplement: Additional file 1 — Sigma-2 ligands enhance gemcitabine induced apoptosis evidenced by detection of Annexin-V by flow cytometry. Panc02 cells were pre-treated with gemcitabine (500 nM) or vehicle for 24 hours prior to exposure to sigma-2 ligands (25 μM) or vehicle for 18 hours. Cells were then stained with Annexin-V-FITC conjugated antibody and propidium iodide (PI) to be assessed by flow cytometry. Sigma-2 ligands induced apoptosis alone and enhanced gemcitabine induced apoptosis. Annexin-V+/PI- represent early apoptotic cells and Annexin-V+/PI+ cells show late apoptotic cells. Data is representative of triplicate experiments. [file 1476-4598-9-298-S1.PPTX]
